# Should Medical Practitioners Dispense Medicines?

**Published:** 1921-01-15

**Authors:** 


### Should Medical Practitioners Dispense Medicines?

We have previously expressed the opinion that medical officers at institutions should not be expected to do dispensing work, and that it is highly desirable that a duly qualified dispenser should perform and be wholly responsible for these duties.

The general question of dispensing by medical practitioners has received a good deal of notice, especially in the lay Press, since the "Kidwelly" case, and in most cases it is held to be highly desirable that the functions of prescribing and dispensing should be distinctly separated. There should be no practical difficulty in this, except, perhaps, in the case of rural areas and scattered districts where there is no practising pharmacist.

### The Insurance Act Procedure.

Since the inception of the National Health Insurance Act all medicines required for insured persons are supplied by pharmacists on the list of "panel" chemists on presentation of the prescription given by the "panel" practitioner in the prescribed form; payment is made direct to the chemist by the Insurance Committee for the area.

In a few rural areas the "panel" doctor has special permission to supply medicines owing to the fact that no pharmacist is available within reasonable distance.

Under the Pharmacy Act, all prescriptions containing "scheduled" poisons must be recorded in a special book, and the evidence of this book kept by a third party is highly advantageous both on general grounds and in the interest of doctor, patient, and chemist. We believe that in most countries outside Great Britain prescribing and dispensing are recognised as distinct functions, to be undertaken only by those specially trained in them—viz.: the doctor and pharmacist—and that a large body of public opinion is averse from continuing the custom obtaining in many districts in this country whereby the doctor dispenses medicines for his own patients and may also sign up the certificate of death. From the medical practitioner's standpoint it is argued that some chemists are in the habit of "prescribing" remedies for certain ailments, and that if all dispensing is to be placed in the hands of chemists, the latter should agree to discontinue any form of "counter prescribing."

### Investigation Needed.

It might be a good thing if the Ministry of Health set up a Committee to go into the whole matter and report on it, as suggested recently by the Pharmaceutical Society, with a view to placing the subject on a more satisfactory basis.

The question of regulating the qualifications of persons acting as "dispensers" at institutions might well be included in the terms of reference, for at present, although qualified pharmacists are employed at most hospitals and institutions, no *legal* qualification is required for those acting as "dispensers."

